# PP2A phosphatase inhibition is anti-fibrotic through Ser77 phosphorylation-mediated ARNT/ARNT homodimer formation

**DOI:** 10.1038/s41598-021-03523-1

**Published:** 2021-12-15

**Authors:** Gunsmaa Nyamsuren, Gregor Rapp, Hassan Dihazi, Elisabeth M. Zeisberg, Desiree Tampe, Björn Tampe, Michael Zeisberg

**Affiliations:** 1 Department of Nephrology and Rheumatology, Göttingen University Medical Center, Georg August University, Göttingen, Germany; 2 Department of Cardiology and Pneumology, Göttingen University Medical Center, Georg August University, Göttingen, Germany; 3 German Center for Cardiovascular Research (DZHK), Robert Koch Street 40, Göttingen, Germany

**Keywords:** Nephrology, Kidney

## Abstract

Aryl hydrocarbon receptor nuclear translocator (ARNT) mediates anti-fibrotic activity in kidney and liver through induction of ALK3-receptor expression and subsequently increased Smad1/5/8 signaling. While expression of ARNT can be pharmacologically induced by sub-immunosuppressive doses of FK506 or by GPI1046, its anti-fibrotic activity is only realized when ARNT-ARNT homodimers form, as opposed to formation of ARNT-AHR or ARNT-HIF1α heterodimers. Mechanisms underlying ARNTs dimerization decision to specifically form ARNT–ARNT homodimers and possible cues to specifically induce ARNT homodimerization have been previously unknown. Here, we demonstrate that phosphorylation of the Ser77 residue is critical for ARNT–ARNT homodimer formation and stabilization. We further demonstrate that inhibition of PP2A phosphatase activity by LB100 enhances ARNT–ARNT homodimers both in vivo and in vitro (mouse tubular epithelial cells and human embryonic kidney cells). In murine models of kidney fibrosis, and also of liver fibrosis, combinations of FK506 or GPI1046 (to induce ARNT expression) with LB100 (to enhance ARNT homodimerization) elicit additive anti-fibrotic activities. Our study provides additional evidence for the anti-fibrotic activity of ARNT–ARNT homodimers and reveals Ser77 phosphorylation as a novel pharmacological target to realize the therapeutic potential of increased ARNT transactivation activity.

## Introduction

Aryl hydrocarbon receptor nuclear translocator (ARNT) is a transcription factor of the family of basic helix-loop-helix-Per-ARNT-Sim (bHLH-PAS) proteins, which elicits its transcription factor activity upon dimerization^1,2^. Depending on its dimerization partner, ARNT is involved in a wide range of biological processes^3^. ARNT was originally discovered as dimerization partner of Aryl hydrocarbon receptor (AHR)^1^. AHR-ARNT heterodimers bind to xenobiotic response elements (XRE) which control expression of numerous detoxification genes^4,5^. Another ARNT dimerization partner is HIF1α, the major mediator of hypoxia responses^6^. Upon reduced oxygen tension HIF1α becomes stabilized and heterodimerizes with ARNT (referred to as HIF1β in association with HIF1α), and the ensuing HIF1 heterodimer then binds to hypoxia response elements (HRE) to induce transcription factors involved in adaptation to hypoxia^7,8^. In addition to its well-known functions when partnering within heterodimers, ARNT can also homodimerize^9^. ARNT homodimers can bind to a palindromic E-box 5′-CACGTG-3′ Arnt/Arnt binding sequence and induce distinct transcriptional responses^9–12^.

ARNT is relevant with regard to kidney disease, because ARNT homodimers induce expression of the type I BMP-receptor ALK3, subsequently attenuating fibrosis in the kidney and the liver^13^. This protective ARNT-dependent activity can be pharmacologically induced by FK506 or GPI1046, which enhance ARNT expression via disruption of a transcriptional FKBP12/YY1 repressor complex^13^. Increased expression of ARNT leads to increased accumulation of ARNT homodimers and subsequently augmented ALK3 expression and protective Smad1/5/8 signaling^13,14^. While both FK506 and GPI1046 induce ARNT expression levels, mechanisms underlying enhanced homodimerization of ARNT as opposed to its heterodimerization are not yet known^15^. We here aimed to gain further insights into mechanisms determining ARNT dimerization decisions to form homodimers and its implications for kidney fibrosis. We demonstrate that ARNT homodimerization is determined by phosphorylation of its Ser77 residue. We further demonstrate that formation of ARNT homodimers can be enhanced by the PP2A phosphatase inhibitor LB100, and that administration of LB100 has an additive protective effect to FK506/GPI1046 in murine models of kidney and liver fibrosis.

## Results

### PP2A phosphatase inhibition increases ARNT homodimers

**Figure 1:**
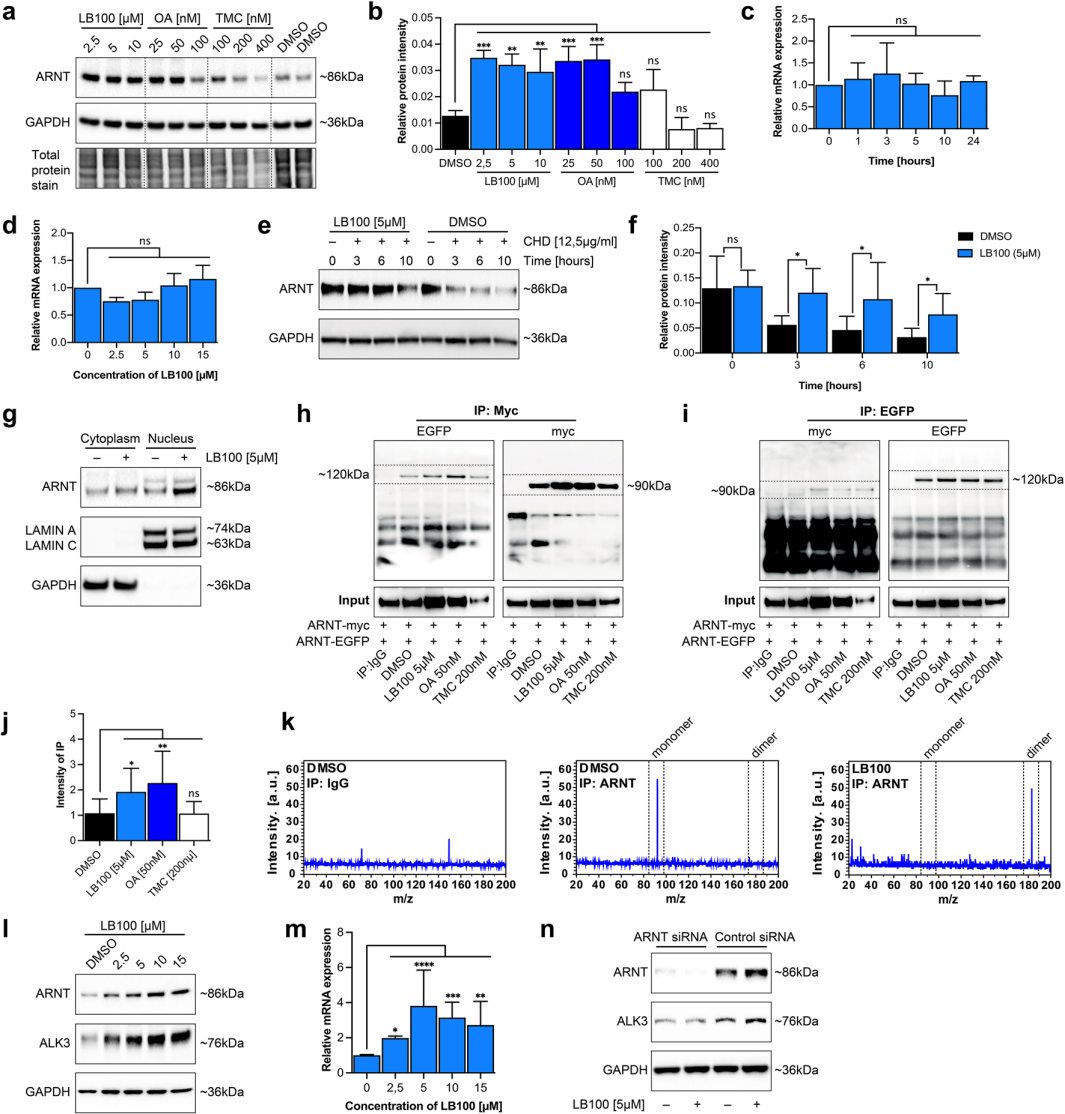
PP2A inhibitor LB100 enhances ARNT homodimer formation. (**a**) ARNT protein level in MCT cells indicated different concentrations following treatment with LB100, OA and TMC relative to control DMSO treated cells. Cells were treated with each phosphatase inhibitor for 3 h. For immunoblotting analysis, GAPDH and total protein stain were used as a loading control. (**b**) Quantification of relative ARNT protein intensity display as graph after normalization of total protein stain. The average mean of the results was obtained from three independent experimental replicates. *p < 0.05; **p < 0.01; ***p < 0.001, 1-way ANOVA versus DMSO treated control cells. Data are shown as mean ± SD. (**c**) Relative expression level of *ARNT* in MCT cells is determined by qRT-PCR after 5 µM of LB100 treatment at indicated time points. Relative mRNA levels were determined after normalization to *GAPDH*, and data are shown as the fold change compared with DMSO treated control cells. The average mean of the results was obtained from three independent experimental replicates. *p < 0.05, 1-way ANOVA versus DMSO treated control cells. (**d**) qRT-PCR was used to determine the mRNA level of *ARNT* in various concentrations of LB100 treatment for 4 h. Relative mRNA levels were determined after normalization to *GAPDH*, and data are shown as the fold change compared with DMSO treated control cells. The average mean of the results was obtained from three independent experimental replicates. *p < 0.05, 1-way ANOVA versus DMSO treated control cells. (**e**) ARNT protein degradation in MCT cells at indicated time points of cycloheximide (CHD) exposure following sub treated with LB100 relative to control DMSO treated cells. For immunoblotting analysis, GAPDH was used as a loading control. (**f**) Quantification of relative ARNT protein intensity display as graph after normalization of GAPDH. The average mean of the results was obtained from three independent experimental replicates. *p < 0.05, 1-way ANOVA versus DMSO treated control cells. Data are shown as mean ± SD. (**g**) Immunoblotting analysis of ARNT expression in cytoplasmic and nuclear fractions of MCT cells treated with LB100 for 4 h. GAPDH and Lamin A/C serve as loading controls for cytoplasmic and nuclear fractions. (**h**, **i**) Representative immunoblotting for co-immunoprecipitation of ARNT-myc and ARNT-EGFP in HEK293 cells treated with control DMSO, LB100 (5 µM), Okadaic acid (OA; 50 nM) and Tautomycetin (TMC; 200 nM) for 4 h. n = 3. (**j**) Intensity of co-immunoprecipitation display as graph after compared with DMSO treated control cells. The average mean of the results was obtained from three independent experimental replicates. *p < 0.05; **p < 0.01, 1-way ANOVA versus DMSO treated cells. Data are shown as mean ± SD. (**k**) The acquisition of the m/z was performed on a Rapiflex MALDI-Tissuetyper mass spectrometer equipied with smartbeam 3D laser in linear mode with sinapinic acid as matrix. In DMSO control samples the mass spectra display a peak with m/z 90 kDa representing the ARNT monomer. In contrast, the mass spectra from LB100 treated samples display a peak with m/z 190 kDa corresponding to ARNT homodimer; n = 3. (**l**). Immunoblotting analysis showing the accumulation of ARNT protein upon various concentrations of LB100 treatment increases ALK3 expression. MCT cells were treated with LB100 for 4 h. For immunoblotting analysis, GAPDH was used as a loading control. (**m**) qRT-PCR was used to determine the mRNA level of *ALK3* in different concentrations of LB100 treatment for 4 h. Relative mRNA levels were determined after normalization to *GAPDH*, and data are shown as the fold change compared with DMSO treated control cells. The average mean of the results was obtained from three independent experimental replicates. *p < 0.05; **p < 0.01; ***p < 0.001; ****p < 0.0001, 1-way ANOVA versus DMSO treated control cells. (**n**). Immunoblotting analysis showing LB100 does not increase ALK3 expression upon ARNT depletion. MCT cells were treated with 5 µM LB100 for 4 h. For immunoblotting analysis, GAPDH was used as a loading control.

Phosphorylation is a fundamental mechanism to regulate transcription factor activity, as it can modulate translocation into the nucleus, protein–DNA binding and protein–protein interactions^16,17^. ARNT is known to be subject to phosphorylation of serine residues, and ARNT transactivation activity on the palindromic E-box 5′-CACGTG-3′ Arnt/Arnt binding sequence is enhanced upon phosphatase inhibition with okadaic acid (OA)^9–11,18^. To gain further insight into mechanisms of phosphorylation in control of ARNT transcription factor activity, we exposed mouse tubular epithelial cells (MCT) to media supplemented with either OA, an inhibitor of type 1 (PP1) and type 2A (PP2A) phosphatases^19^, the PP1 inhibitor tautomycetin (TMC)^20^, or with the PP2A inhibitor LB100 at pre-established nontoxic doses (Supplementary Fig. S1)^21^. Exposure to OA (PP1 and PP2A inhibition) or LB100 (selective PP2A) inhibition induced ARNT protein levels, whereas TMC (selective PP1 inhibition) had no effect, suggesting a role for PP2A-dependent dephosphorylation in control of ARNT (Fig. 1a,b). Because ARNT accumulation upon PP2A inhibition was independent of *ARNT* mRNA expression (Fig. 1c,d), we performed cycloheximide chase assays. In absence of protein translation, ARNT protein depletion was significantly slower when PP2A activity was blocked by LB100 (Fig. 1e,f). These experiments revealed that PP2A phosphatase inhibition leads to increased ARNT protein levels because the degradation process is impaired. As the biological activity of ARNT is elicited through its transcription factor activity, we next thought to gain insight into the subcellular distribution of accumulated ARNT upon LB100 exposure. Fractionation of nuclear and cytoplasmic proteins revealed that upon LB100-mediated PP2A phosphatase inhibition, ARNT predominantly accumulated within the nucleus (Fig. 1g).

ARNT elicits its transcription factor activity upon homodimerization. Furthermore, because assembly into dimers is a principal mechanism through which phosphorylation increases resistance of proteins to proteolytic degradation, we next elucidated the impact of pharmacological PP2A inhibition on ARNT dimer accumulation. For this purpose, we co-transfected HEK293 cells with expression plasmids encoding for EGFP-tagged and myc-tagged ARNT and supplemented media with OA (PP1 and PP2A inhibition), LB100 (PP2A inhibition) or TMC (PP1 inhibition) at pre-established doses. Immunoprecipitation with myc-antibodies and subsequent immunoblotting revealed increased pull-down of EGFP-tagged ARNT upon OA or LB100 treatment, suggesting that ARNT homodimerization is increased upon PP2A inhibition (Fig. 1h–j). To further explore impact of PP2A inhibition on ARNT homodimerization within MCT cells, we performed immunoprecipitation using ARNT antibody and subsequent mass spectrometer analysis to measure the ARNT protein weight of endogenous ARNT. While the measured molecular weight of ARNT from the control cells peaked at m/z around 90 kDa (corresponding to monomeric ARNT), ARNT weight of LB100 treated cells peaked at m/z around 180 kDa (corresponding with dimeric ARNT, Fig. 1k). Because reno-protective activity of ARNT is mediated by increased ALK3 expression (through ARNT homodimers binding to the palindromic E-box 5′-CACGTG-3′ Arnt/Arnt binding sequence within the proximal ALK3 promoter), we next assessed whether LB100-induced ARNT homodimerization is sufficient to enhance ALK3 expression^13^. Exposure of cultured MCT cells to LB100 induced ALK3 expression in dose-dependent manner, correlating with enhanced ARNT levels (Fig. 1l). Accumulation of ALK3 protein correlated with increased *ALK3* mRNA expression levels, suggesting that observed ALK3 increased in response to LB100 is due to increased expression, and not due to possible inhibition of proteolytic degradation (Fig. 1m). To confirm that observed induction of ALK3 expression in response to LB100 is indeed mediated by ARNT, we performed control experiments in which MCT cells had been transfected with either ARNT siRNA or control scrambled siRNA. Upon depletion of ARNT, LB100 does not induce ALK3 accumulation (Fig. 1n), providing a causal link between LB100-mediated PP2A inhibition, nuclear accumulation of ARNT homodimers and increased ALK3 expression.

### Serine 77 phosphorylation of ARNT is required for ARNT homodimerization

**Figure 2:**
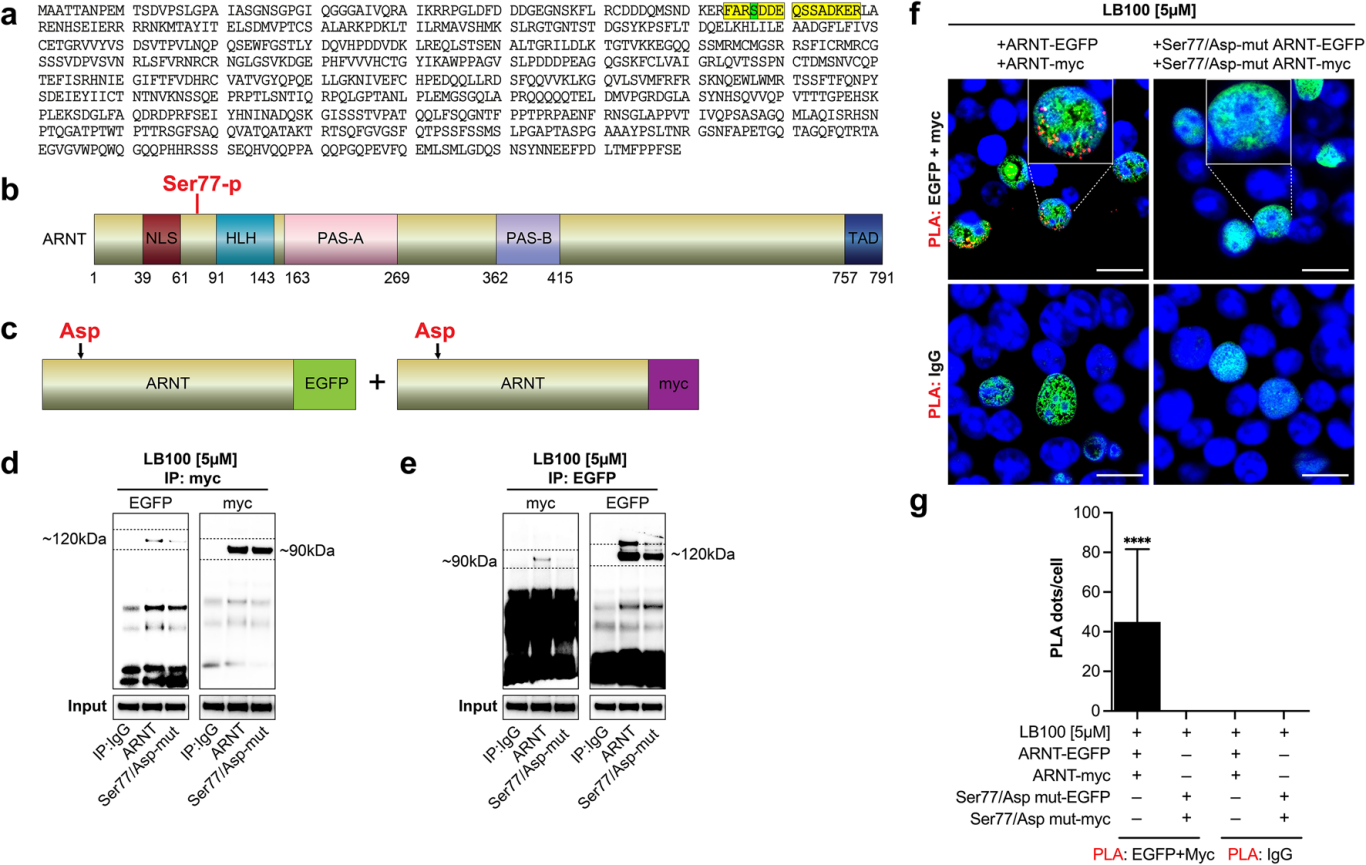
Serine77 phosphorylation site of ARNT is responsible for the homodimer formation. (**a**) ARNT phosphorylation site is identified at the serine on position 4 of the peptide (Ser77 in ARNT) after 5 µM of LB100 treatment. (**b**) Domain organization of ARNT (NLS-nuclear localization domain; bHLH-basic helix loop helix; PAS-periodicity/ARNT/single-minded; TAD-transactivation domain). (**c**) EGFP or myc-tagged wild type ARNT overexpression constructs in which serine 77 was changed to the aspartic acid residue. (**d**, **e**) Representative immunoblotting for co-immunoprecipitation of EGFP or myc-tagged wildtype and mutated ARNT expression construct after 4 h of LB100 treatment (5 µM). (**f**) Representative confocal images of proximity ligation assay (PLA) for EGFP and myc-tagged wild type and mutated ARNT expressing HEK293 cells after LB100 (5 µM) treatment. Scale bars: 15 µm. (**g**) Quantitative analysis showing PLA signals, measured as dots per cells (N = 20 cells). ****p < 0.0001, 1-way ANOVA versus IgG. Data are shown as mean ± SD.

To gain further insights into the role of phosphorylation in ARNT homodimerization we next performed ARNT immunoprecipitation of LB100-treated, ARNT-overexpressing HEK293 cells and subsequent mass spectrometric analysis of the phosphopeptide-enriched fraction from ARNT immunoprecipitation digests. Upon LB100 treatment, we identified a single differentially phosphorylated peak. The identified phosphopeptide FARpSDDEQSSADKER corresponded to the ARNT sequence in the position 74–88 and the observed fragment ions identified the serine on position 4 in the peptide (Ser77) as ARNT phosphorylation site (Fig. 2a, Supplementary Fig. S2). In summary, mass spectrometric analysis of the phosphopeptide-enriched fraction identified the Ser77 to be selectively phosphorylated in LB100-induced ARNT homodimers (Fig. 2b).

To assess the functional importance of phosphorylation of Ser77 in ARNT homodimer formation, we performed site-directed mutagenesis to change Ser77 to the negatively charged aspartic acid residue in previously established myc-tagged ARNT and EGFP-tagged ARNT constructs (Fig. 2c). We transfected cells with expression plasmids encoding either for myc-tagged and EGFP-tagged wildtype ARNT or for Ser77/Asp-mutant ARNT and performed immunoprecipitation and immunoblotting upon LB100 treatment. We did not detect pull-down of Ser77/Asp-mutant ARNT (Fig. 2d,e), providing evidence that ARNT homodimerization is not by charge, but specifically by addition of a phosphoryl-group to Ser77. To further confirm a critical role of Ser77 phosphorylation in ARNT homodimerization we performed proximal ligation assay on HEK293 cells which had been co-transfected with expression plasmids encoding either for myc-tagged, EGFP-tagged wildtype ARNT or for Ser77/Asp-mutant ARNT. Signals indicative of co-localization positive signals were not detectable in cells which had been transfected with Ser77/Asp-mutant ARNT constructs, further confirming an essential role of Ser77-phosphorylation for the formation of ARNT homodimers (Fig. 2f,g).

### LB100 synergizes with FK506/GPI1046 to increase ARNT homodimerization and to attenuate fibrosis in murine UUO kidneys

**Figure 3:**
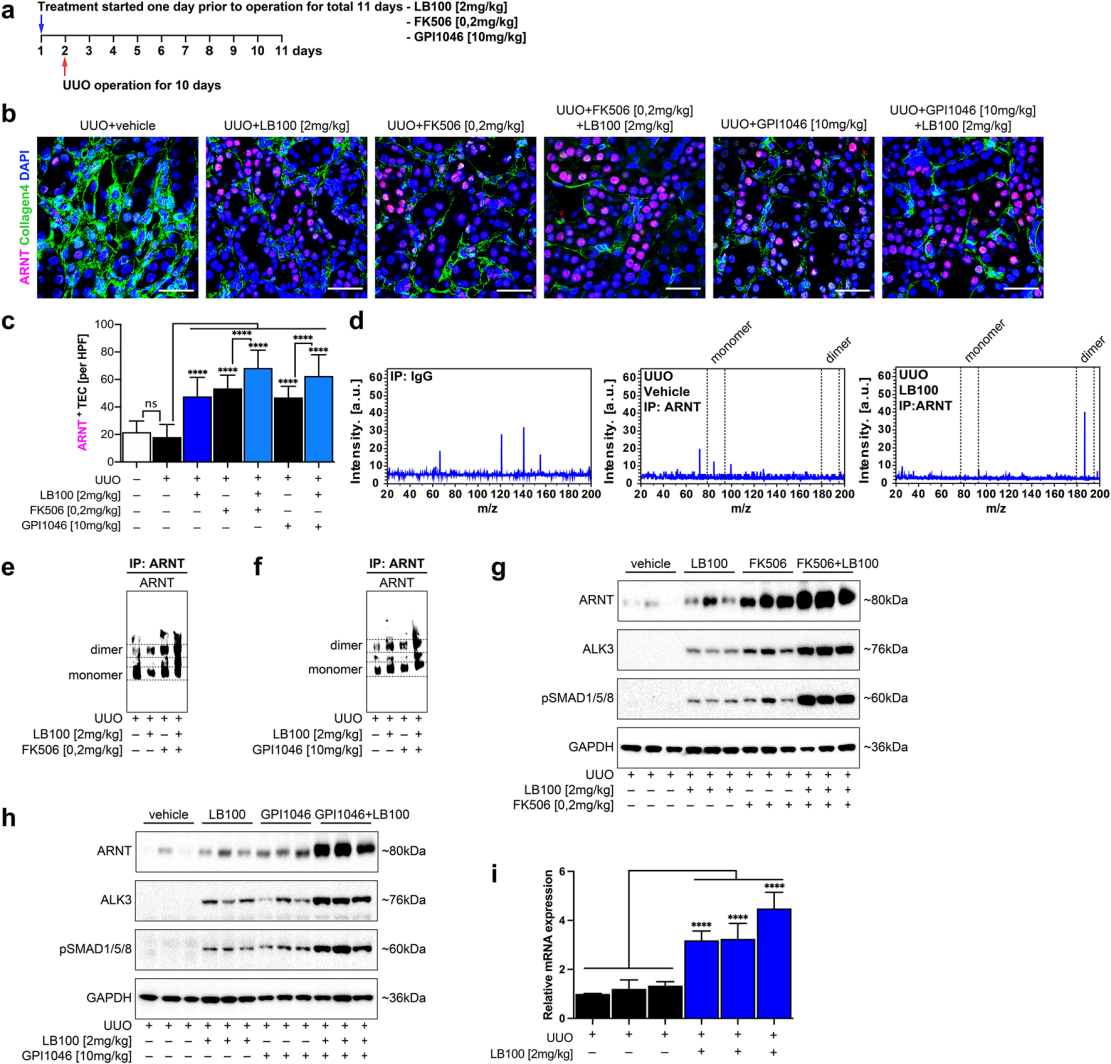
LB100 induces ARNT homodimerization in the obstructed mice kidney and enhances ALK3 signaling axis. (**a**) UUO challenged mice were treated with vehicle buffer (5% glucose), LB100 (2 mg/kg subcutaneously per day), FK506 (0.2 mg/kg orally per day), GPI1046 (10 mg/kg subcutaneously per day) or indicated combinations at 24 h before surgery. (**b**) Immunofluorescence labeling for ARNT (red) in fibrotic mice sections, indicating the expression of ARNT in each group. n = 6/group. Scale bars: 15 µm. (**c**) Quantitative analysis showing ARNT positive nucleus per tubule in each group. ****p < 0.0001, 1-way ANOVA versus vehicle group. Data are shown as mean ± SD. (**d**) The acquisition of the m/z of the endogenous ARNT pull down in total kidney lysates was carried out using the Rapiflex MALDI-Tissuetyper mass spectrometer equipped with smartbeam 3D laser. In the vehicle samples, the mass spectra display a weak peak with a m/z between 75 and 85 kDa representing the ARNT monomer. In contrast, the mass spectra from LB100 treated mice kidneys show a peak with m/z 190 kDa corresponding to ARNT homodimer. (**e**, **f**) Native protein conformation representing ARNT homodimerization upon LB100, FK506 and GPI1046 administration in total kidney lysates after endogenous ARNT pull-down. (**g**, **h**) Representative immunoblotting analysis of ARNT protein levels in the obstructed mice kidney in each experimental group. Increased activation of ALK3 signaling was observed in the treated groups. For immunoblotting analysis, GAPDH was used as a loading control. (**i**) qRT-PCR was used to determine the mRNA level of *ALK3* in LB100 exposed obstructed mice kidneys. n = 3 in each group. Relative mRNA levels were determined after normalization to *GAPDH*, and data are shown as the fold change compared with vehicle group. ****p < 0.0001, 1-way ANOVA versus vehicle group.

Our studies demonstrated that PP2A inhibition by LB100 transactivation activity on the palindromic E-box 5′-CACGTG-3′ Arnt/Arnt binding sequence via stabilization of ARNT homodimers through preservation of Ser77 phosphorylation without impacting ARNT expression levels. Because our previous studies had discovered renoprotective ALK3 expression upon FK506/GPI1046-induced ARNT expression, we hypothesized that two distinct mechanisms (increased ARNT expression and increased ARNT homodimerization) could be combined to enhance beneficial ARNT-mediated renoprotection^13^. To test this hypothesis, we treated mice with combinations of LB100 and FK506 or GPI1046 and challenged mice with unilateral ureter obstruction (UUO) 24 h after injections (Fig. 3a). Total ARNT levels were enhanced by each treatment with LB100, FK506 or GPI1046 (Fig. 3b,c,g,h). However, combination of LB100 with either FK506 or GPI1046 further enhanced ARNT levels (Fig. 3b,c,g,h). Combination of LB100 with FK506 or GPI1046 not only further increased total ARNT levels but had also robust additive effects on ARNT homodimer accumulation as was evidenced by immunoprecipitation, native gel electrophoresis and subsequent immunoblotting using ARNT antibody (Fig. 3d–f). Increased ARNT levels upon LB100 treatment correlated with ALK3 accumulation (Fig. 3g–i), which mediates anti-fibrotic activity of BMPs^13,14^. ALK3-induced anti-fibrotic activity is mediated by nuclear translocation of the phosphorylated transcription factors Smad1, Smad5 and Smad8. Assessment of pSmad1/5/8 nuclear translocation through immunolabeling with antibodies specific to the phosphorylated forms revealed increased pSmad1/5/8 signaling activity upon LB100 treatment and also an additive effect to both FK506 (Fig. 3g) and GPI1046 (Fig. 3h). Monotherapy with LB100 significantly induced *ALK3* mRNA expression, suggesting that observed effect of LB100 on ALK3 was due to increased ARNT-mediated *ALK3* expression and not due to reduced ALK3 degradation (Fig. 3i).

**Figure 4:**
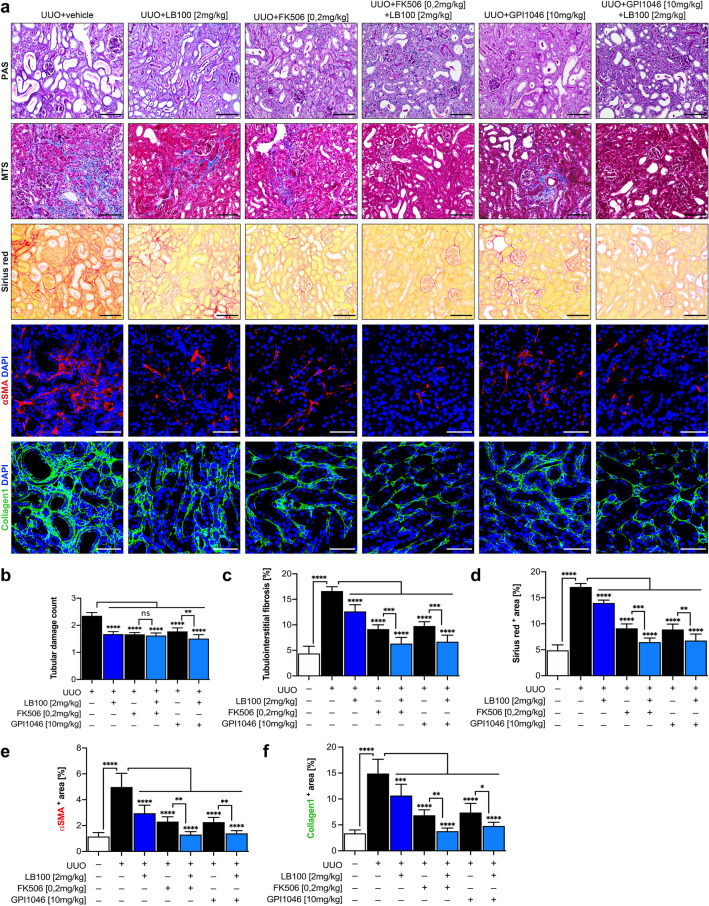
Enhanced formation of ARNT homodimerization effectively protects mice from experimental chronic kidney injury. (**a**) Representative light microscopy images of Periodic acid Schiff (PAS, top), Masson’s trichrome stain (MTS, middle) and Sirius red (bottom) stained sections of the renal cortex from indicated groups. n = 6 in each group. Scale bars: 25 µm. Immunofluorescent labeling for αSMA (red) and Collagen1 (green) staining, indicating the fibrotic area in the renal cortex in each group. Scale bars: 15 µm. (**b**) Quantitative analysis of tubular damage by PAS staining from the indicated group. (**c**–**f**) Graphs of the interstitial fibrotic area by MTS, Sirius red, αSMA and Collagen1 staining in the renal cortex from each group. *p < 0.05; **p < 0.01; ***p < 0.001; ****p < 0.0001, 1-way ANOVA. Data are shown as mean ± SD.

We next aimed to explore whether observed additive effect of LB100 to FK506/GPI1046-induced ARNT homodimer formation and ALK3 expression also correlated with further attenuation of renal fibrosis. Because ALK3-elicits its anti-fibrotic activity in the kidney primarily in tubular epithelial cells, we assessed tubular atrophy in Periodic acid-Schiff (PAS)-stained tissue sections (Fig. 4a,b). To assess a possible impact on interstitial fibrosis, we evaluated the fibrotic area in kidney sections which had been stained with Mason Trichrome Staining (MTS), Sirius Red Staining or with antibodies to type I collagen (Fig. 4a,c,d,f). To assess accumulation of myofibroblasts, we quantified the area stained with antibodies to αSMA (Fig. 4a,e). LB100 reduced tubular injury (Fig. 4b), interstitial fibrosis (Fig. 4c,d), αSMA-positive myofibroblasts (Fig. 4e) and type I collagen deposition (Fig. 4f), and further significantly added to observed beneficial effects of FK506 and GPI1046 treatment (Fig. 4a–f). To control for possible systemic effects of the treatments, major organs, including heart, liver, lung, intestine, brain and control kidney were dissected out, and tissues were subjected to hematoxylin and eosin (HE) staining revealing no gross abnormalities or inflammation in any of the organs and LB100 exposed experimental mice groups (Supplementary Fig. S3).

### LB100 synergizes with FK506/GPI1046 to attenuate experimental liver fibrosis

**Figure 5:**
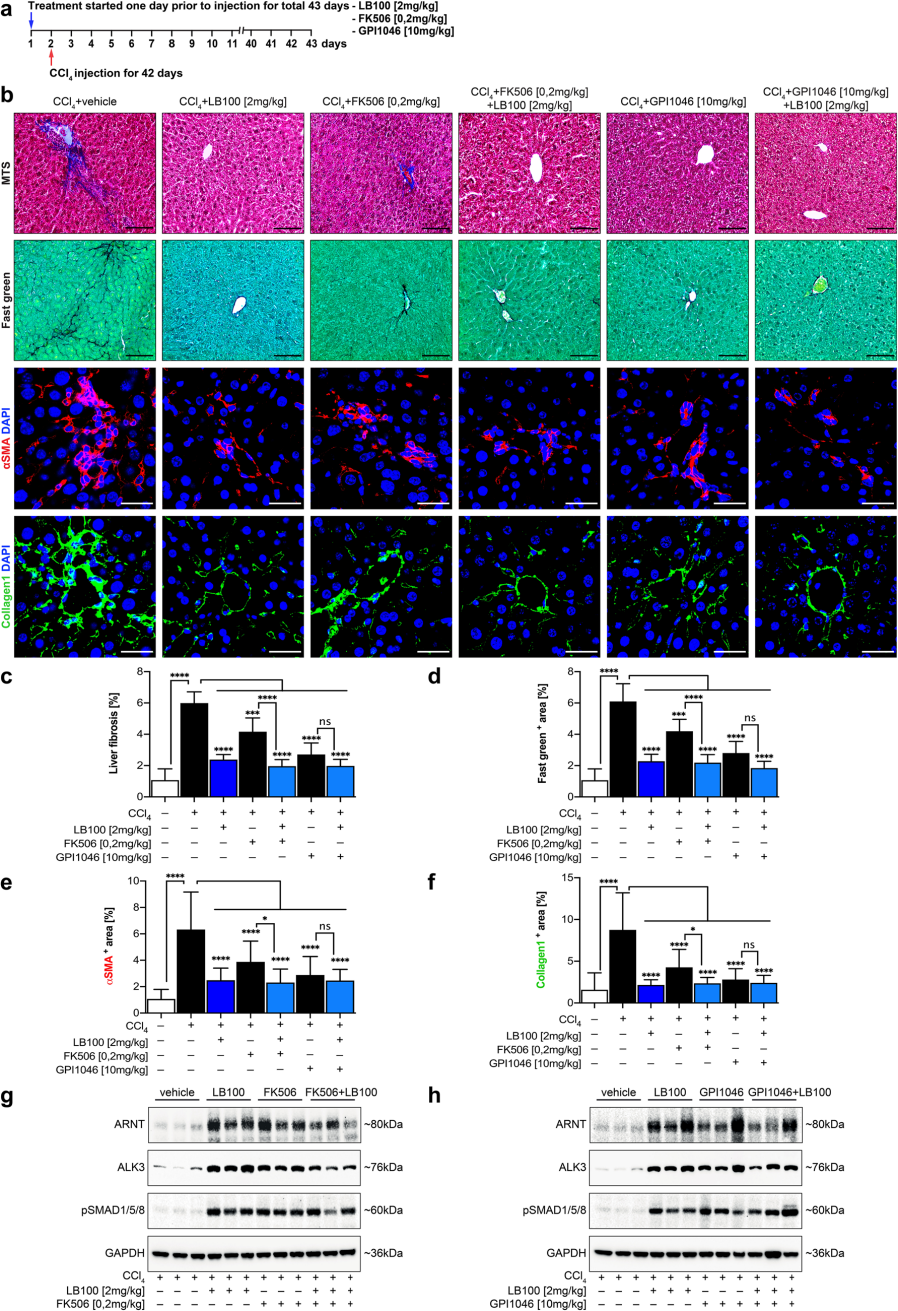
LB100 protects experimental liver injury via the activation of ARNT/ALK3 signaling axis and improves the hepatoprotective role of FK506/GPI1046. (**a**) Mice were received CCl_4_ for 42 days. One day before CCl_4_ injection, mice were treated with vehicle buffer (5% glucose), LB100 (2 mg/kg subcutaneously per day), FK506 (0.2 mg/kg orally per day), GPI1046 (10 mg/kg subcutaneously per day) or indicated combinations simultaneously for 43 days. (**b**) Representative microscopy images of MTS (top), Fast green (middle), αSMA (middle) and Collagen1 (bottom) stained sections of CCl_4_ induced liver fibrotic mice from each group. n = 6/group. Scale bars: 25 µm (MTS, Fast green); 15 µm (αSMA, Collagen1). (**c**–**f**) Quantitative analysis of the fibrotic area in the liver section from the indicated experimental group. *p < 0.05; **p < 0.01; ***p < 0.001; ****p < 0.0001, 1-way ANOVA. Data are shown as mean ± SD. (**g**,** h**) Representative immunoblotting analysis showing the expression level of ARNT, ALK3 and pSmad1/5/8 protein in fibrotic mice liver in each experimental group. Increased activation of ALK3 signaling was observed in the treated groups. For immunoblotting analysis, GAPDH was used as a loading control.

Because our previous studies had identified that FK506/GPI1046-induced ARNT expression not only attenuates fibrosis in the kidney but also in experimental models of liver fibrosis, we next aimed to explore if LB100 was equally beneficial in liver as in kidney^13^. We treated mice with combinations of LB100 and FK506 or GPI1046 and challenged mice with carbon tetrachloride (CCl_4_) 24 h after injections (Fig. 5a). Monotherapy with LB100 reduced fibrosis (Fig. 5b–d), accumulation of αSMA-positive myofibroblasts (Fig. 5e) and of type I collagen (Fig. 5f), and equivalent effect as either FK506, GPI1045 or combination (Fig. 5b–f). Anti-fibrotic activity of LB100 correlated with increased ARNT levels, ALK3 expression and pSmad1/5/8 signaling (Fig. 5g,h). While combinations of FK506/ LB100 and GPI1046/ LB100 were equally effective, the addition of LB100 to GPI1046 failed to reach significance, as compared to GPI1046 monotherapy, even though a positive trend was observed.

## Discussion

Here, we report that inhibition of PP2A phosphatase activity increases intracellular accumulation of ARNT–ARNT homodimers. This effect is dependent on enhanced ARNT–ARNT homodimerization and decreased ARNT proteolytic degradation, but independent of ARNT transcription (which remains unchanged upon PP2A inhibition). We further identify that Ser77 phosphorylation plays a critical role in ARNT homodimerization, as ARNT–ARNT homodimers do not form with Ser77/Asp-mutant ARNT proteins. In light of previous studies which identified anti-fibrotic activity upon increased ARNT expression, we further demonstrate attenuated fibrosis upon monotherapy with the PP2A inhibitor LB100 in liver fibrosis model, and additive anti-fibrotic activities upon combination with pharmacological inducers of ARNT expression FK506 or GPI1046 in murine models of kidney fibrosis.

In line with previous observations, our study suggests that accumulation of LB100-induced ARNT homodimers elicits anti-fibrotic activity through induction of ALK3 expression, which subsequently results in increased protective pSmad1/5/8 signaling and ultimately in attenuated fibrosis^13,14,30^. While LB100 does not induce ALK3 expression when ARNT is depleted, we are aware that additional mechanisms are involved which mediate anti-fibrotic activity of LB100. In this regard, knowledge on the overall contribution of the ubiquitously expressed phosphatase PP2A to kidney fibrosis is relatively scarce. A previous publication reported reduced kidney fibrosis upon specific inhibition of the PPA catalytic subunit through inhibition of endothelial-mesenchymal transition^31^. Furthermore, while there is substantial evidence for the protective role of increased ALK3 expression and pSmad1/5/8 signaling in the kidney, we are aware that additional transcriptional targets of ARNT may contribute to its anti-fibrotic activity.

Our data is in line with a previous study, which reported increased transactivation activity on the palindromic E-box 5′-CACGTG-3′ Arnt/Arnt binding sequence when cells were exposed to the combined PP1/PP2A inhibitor okadaic acid^18^, and we now demonstrate that this increased ARNT transactivation activity is due to increased homodimerization. A possible additive effect on enhanced recruitment of yet to be identified co-transcription factors, nuclear entry, or DNA binding affinity needs to be addressed in future studies.

ARNT is composed of a nuclear localization signal (NLS), a bHLH domain required for DNA binding, two PAS domains (PAS-A and PAS-B), and a TAD domain^22^. Identified critical amino acid Ser77 is located between NLS and bHLH domains and thus in a distinct location from amino acids which were previously demonstrated to mediate heterodimerization into ARNT-HIF1α and ARNT-AHR dimers, which are primarily located within the PAS-A domain^23–25^. As ARNT is involved in numerous biological processes in distinct cell types, such as within kidney epithelial cells, pancreatic islet beta cells or cancer cells^13,26,27^, our studies cannot rule out the possibilities that alternate mechanisms may exist, which regulate ARNT phosphorylation and target gene specificities in different cell types. Furthermore, additional studies are required to explore, whether ARNT Ser77 phosphorylation status (and increased ARNT homodimerization) has an impact on HIF1 formation and hypoxia responses under hypoxic conditions. We are aware of the technical limitations of our studies which are caused by the use of non-specific inhibitors and generation of constitutively phosphorylated Ser77 ARNT may be required to ultimately address these questions in the future.

In extension of previous studies which demonstrated anti-fibrotic activities of increased ARNT expression and subsequently increased BMP-signaling in kidney and liver^13^, our study now demonstrates that enforced ARNT homodimerization through administration of the PP2A inhibitor LB100 also attenuates fibrosis in murine models of kidney and liver fibrosis. Furthermore, administration of LB100 has an additional therapeutic effect when combined with FK506 or GPI1046 which both induce anti-fibrotic activity via induction of ARNT expression. Therapeutic efficacy of LB100 monotherapy provides further evidence that the anti-fibrotic effect of ARNT depends on ARNT–ARNT homodimer transactivation activity, while in our system we did not observe interactions with hypoxia or xenobiotic detoxification pathways. In light of other PAS domain protein-modifying drugs which are undergoing clinical testing, it is attractive to speculate that compounds more specific than LB100 could be equally developed to enhance ARNT homodimerization in the future to attenuate chronic organ failure and to combat fibrosis.

## Methods

### In vivo experimental protocol

Animal care and experimental protocols complied with German animal care and ethics legislation and were approved by the local government authorities (LAVES, Oldenburg, Germany) and the animal licensing committee of Göttingen University Medical Center, Germany in compliance with the ARRIVE guidelines (cite: PMID: 20613859). *C57BL/6* mice used for experiments were 8–12 weeks of age and maintained under the standard condition. Mice were divided into four groups: vehicle control (n = 6), LB100 given (n = 6), the combination of LB100 and FK506 given (n = 6) and those given combinations of LB100 and GPI1046 (n = 6). Unilateral ureteral obstruction (UUO) and carbon tetrachloride (CCl_4_) induced chronic liver injury models were performed as previously described^13^.

### Drug preparation and treatment

FK506 and GPI1046 were prepared and applied as described previously^13^. Briefly, mice were treated orally with 0.2 mg/kg body weight per day FK506 (Abcam Biochemicals, Cambridge, UK) or subcutaneously with 10 mg/kg body weight per day GPI1046 (Santa Cruz Biotechnology, Dallas, USA) with the combination of LB100 once daily starting one day prior of surgery. LB100 (Selleck Chemicals, USA) was prepared by dissolving in sterile PBS at a concentration of 50 mM stock solution. One day before surgery, mice were injected subcutaneously with 2 mg/kg body weight per day LB100. Okadaic acid (Cell signaling technology, USA) 1 mM stock solutions were prepared by dissolving in DMSO. Tautomycetin (Tocris, USA) was supplied as pre-dissolved in DMSO at a concentration of 10 mg/ml.

### Cell culture and transfection

HEK293 cell line is derived from human embryonic kidney cell, purchased from American Type Culture Collection (ATCC clone CRL-1573, USA). The murine proximal TEC line MCT was stabilized from proximal tubular epithelial cells of SJL mice (laboratory stock, derived from Neilson’s laboratory)^28^. Both cells were cultured in Dulbecco’s modified Eagle’s medium (DMEM, Gibco, USA) supplemented with 10% fetal bovine serum (FBS, Gibco, Germany) and 1% penicillin–streptomycin (PS, Gibco, USA) at 37 °C in 5% CO_2_. Cell lines were routinely tested for mycoplasma contamination. For phosphatase inhibitor treatment, 1.5–2 × 10^5^ or 8 × 10^6^ MCT cells were seeded in one well of a 6-well culture plate or 10 cm plates in antibiotic free normal medium. After 24 h, cells were treated with LB100 (Selleck Chemicals, USA), Okadaic acid (Cell signaling technology, USA) or Tautomycetin (Tocris, USA) at indicated concentrations or time. In general, cells were harvested for further analysis after 3–4 h of treatment. For transfection 4 × 10^6^ HEK293 cells were seeded in 10 cm culture plates one day before transfection using medium without antibiotics. Two hours before transfection the medium (without antibiotics) was exchanged. Total 30 µg of EGFP and myc-tagged ARNT control or mutated constructs were transiently transfected to HEK293 cells using Lipofectamine 2000 transfection reagent (Invitrogen, USA) according to the manufacturer’s instructions. After 6 h of incubation, transfection medium was replaced by normal medium. After 48 h of transfection, cells were treated with phosphatase inhibitors for 4 h. For knockdown experiments, 2 × 10^5^ MCT cells were seeded in one well of a 6-well culture plate and transfected with 80 pmol mARNT siRNA (Santa Cruz Biotechnology, USA) or scrambled siRNA (Santa Cruz Biotechnology, USA) using Lipofectamine 2000 transfection reagent (Invitrogen, USA).

### Cytotoxicity assay

Cell viability was assessed with MTT assay (Roche) according to the manufacturer’s instructions. Approximately 3 × 10^3^ cells were seeded into each well of 96-well plates. After overnight culture in complete medium, cells were treated with different concentrations of the drugs over different time points. Absorbance values were measured at 550 nm (nm) wavelength with Synergy 2 Multi-Detection Microplate Reader spectrophotometer (BioTek).

### Cycloheximide chase experiment

Tubular epithelial cells were treated with 12.5 µg/ml cycloheximide (Sigma-Aldrich, Germany) with or without 5 µM LB100. Cells were collected and lysed for western blotting analyzing after 3, 6 and 10 h of incubation with cycloheximide.

### Immunoblotting

Western blot analysis was carried out on total protein extracts from mouse tissues or cultured cells. Frozen tissues were homogenized by TissueLyser LT (Qiagen Inc, Germany) in NP40 Cell Lysis Buffer (Invitrogen, USA) containing phosphatase (Sigma-Aldrich, USA) and protease inhibitor (Roche, Switzerland) followed by sonication and centrifugation at 12,000×*g* for 20 min at 4 °C. Proteins were extracted from the cultured cells by NP40 Cell Lysis Buffer (Invitrogen, USA) containing phosphatase (Sigma-Aldrich, USA) and protease inhibitor (Roche, Switzerland) followed by sonication and centrifugation at 12,000×*g* for 20 min at 4 °C to clear the lysates. The supernatant was collected for immunoblotting. Samples containing 10 µg of extracted proteins were resolved by NuPAGE SDS-PAGE (Novex Life technologies, USA). Native protein conformation and protein complex were quantified by Blue Native PAGE technique. Native protein samples were prepared by NativePAGE Sample Prep Kit (Invitrogen, USA) and were separated by NativePAGE Novex Bis–Tris gels (Novex Life technologies, USA). Nuclear and cytoplasmic proteins were fractionated using NE-PER Nuclear and Cytoplasmic Extraction Reagents (Thermo Scientific, USA) kit and following the manufacturer’s detailed instructions. Blots were often stripped with Thermo Scientific Restore PLUS Western Blot Stripping Buffer (Thermo Fisher Scientific, Germany) for reprobing. The following antibodies were used for immunoblotting: ALK3 (1:1000; 38–6000, Invitrogen, USA), ARNT (1:1000; 5537, Cell Signaling Technology, USA), GAPDH (1:8000; sc-32233, Santa Cruz, USA), Lamin A/C (1:4000; 4777, Cell Signaling Technology, USA) and pSmad158 (1:000; 13,820, Cell Signaling Technology, USA). Proteins were detected by LumiGLO chemiluminescent system (Cell Signaling Technology, USA) with appropriate secondary antibodies: anti-mouse HRP (1:2500; Dako, Denmark) and anti-rabbit HRP (1:2500; 7074, Cell Signaling Technology, USA) using ChemiDoc MP Imaging System (Bio-Rad, USA). Antibodies were listed in the Supplementary Table S2.

### Immunoprecipitation

After 4 h of phosphatase inhibitors treatment cells were lysed with Protein G Immunoprecipitation Kit (Roche, Switzerland) according to the manufacturer’s instructions. Briefly cells were lysed in 1 ml Lysis buffer/wash buffer 1 (Protein G Immunoprecipitation Kit; Roche, Switzerland) containing phosphatase (Sigma-Aldrich, USA) and protease inhibitor (Roche, Switzerland) for 1 h at 4 °C with constant agitation. After centrifugation at 12,000×*g* for 10 min at 4 °C, supernatants were incubated with protein A/G PLUS Agarose beads (Santa Cruz, USA) for the lysate pre-cleaning (1 h at 4 °C) and pull-down (overnight at 4 °C). ARNT (5537, Cell Signaling Technology, USA), EGFP (MA5-15256, Invitrogen, USA), myc-tag (2276; Cell Signaling Technology, USA) and mouse IgG (Santa Cruz, USA) antibodies were used for immunoprecipitation. Captured immunocomplexes were immunoblotted with ARNT (1:1000; 5537, Cell Signaling Technology, USA), EGFP (1:1000; MA5-15256, Invitrogen, USA) and myc-tag (1:1000; 2276; Cell Signaling Technology, USA) antibodies. For tissue immunoprecipitation, 3–5 mg piece of kidney tissues were homogenized in 300 µl NP40 Cell Lysis Buffer (Invitrogen, USA) containing phosphatase (Sigma-Aldrich, USA) and protease inhibitor (Roche, Switzerland) and further lysed with additional 500 µl lysis buffer for 2 h at 4 °C with constant agitation. After centrifugation at 12,000×*g* for 20 min at 4 °C, supernatants were incubated with ARNT (5537, Cell Signaling Technology, USA) or rabbit IgG (Invitrogen, USA) conjugated Dynabeads Protein G (Invitrogen, USA) complex. The beads were then washed with washing buffer of Dynabeads Protein G Immunoprecipitation Kit (Invitrogen, USA) and protein complex were eluted in 0.3% trifluoroacetic acid (TFA; Sigma AIdrich, USA) and immediately mixed with equal volume of ammonium acetate buffer (100 mM, pH 9) resulting in 5–8 µM ARNT. Liquid native MALDI mass spectrometry using Rapiflex MALDI Tissuetyper equipped with smartbeam 3D laser was utilized to investigate ARNT-dimerization^29^. The technique encompasses methods to keep noncovalent interactions of biomolecular complexes intact in the gas phase throughout the instrument and to measure the mass-to-charge ratios protein complexes directly in the mass spectrometer. The eluted samples were diluted 50% with glycerol and then mixed 1:1 on-stage with 1 μl of matrix solution. The analyte-matrix samples were spotted onto a stainless-steel sample stage with final concentrations of protein complexes in the deposits in the 1–2 μM range. Protein standards were spotted using the same methods as samples. Linear positive ion mode by accumulation of 200–1000 laser shots. Calibration was achieved using the Protein Standard II (Burker Bremen, Germany) supplied with high purity IgG.

### Histological analysis

Tissue samples were fixed in 4% formaldehyde for overnight, dehydrated in a graded ethanol series, embedded in paraffin for sectioning (3 µm) and stained with hematoxylin and eosin stain (HE), Periodic acid-Schiff (PAS), Masson’s trichrome stain (MTS) and Sirius Red/Fast green. The images were captured by cellSens (Olympus, Japan). The PAS-stained sections were analyzed using a random grading method according to a semi-quantitative score of 0–3 at 200 ×magnification in a total number of 100 tubules per mouse section. For each slide, the presence of tubular damage was documented. Interstitial fibrosis was quantified in MTS (400× magnification) and Sirius Red/Fast green (400× magnification) stained section and images were captured in ten random visual fields per mouse and the mean area of positive staining was evaluated by using a 10 mm^2^ graticule.

### Immunofluorescence

Paraffin-embedded mice sections were subjected to immunofluorescence as following, deparaffinized, antigen retrieval and antibody incubation. The following primary antibodies were used: ARNT (1:100; 5537, Cell Signaling Technology, USA), Collagen1 (1:10; 1310-30, Southern Biotech, USA), αSMA (1:500; A5228, Sigma, USA) were used. Antibodies were listed in the Supplementary Table S2. The following secondary antibodies were used: Alexa Fluor 488 or 568 (1:250; Life Technologies, USA). The nuclei were stained with 4,6-diamidino-2-phenylindole (DAPI; Vector Laboratories, USA). Pictures were captured by confocal microscopy.

### Proximity ligation assay (PLA)

PLA was performed with the mouse/rabbit Duolink In Situ Red starter Kit (Olink, Sweden). Approximately 5 × 10^2^ HEK293 cells were seeded in per well of an 8-well chamber slide (Falcon, USA). The cells were then transiently co-transfected with EGFP and myc-tagged wild type ARNT or Ser77/Asp-mutant ARNT constructs. After 48 h of transfection cells were treated with 5 µM LB100 for 4 h and fixed in absolute methanol for 20 min at − 20 °C. The fixed cells were then permeabilized with 0.1% Triton X-100 (Sigma Aldrich, USA) for 15 min at room temperature and incubated in the blocking buffer of the kit for 1 h at 37 °C in a humidified chamber. The cells were then incubated with EGFP (1:100; MA5-15256, Invitrogen, USA) and myc-tag (1:100; 2276; Cell Signaling Technology, USA) antibodies for 2 h at room temperature. For the rest of the instruction, the manufacturer’s protocols were followed. PLA data from confocal microscopy images were analyzed by Image J Software.

### Quantitative real-time PCR analysis

Total RNA was extracted by the TRIzol reagent (Life technologies, USA) and purified by PureLink RNA Mini Kit (Ambion, USA). 500 ng of total RNAs were used for cDNA synthesis using the SuperScript II Reverse Transcriptase kit (Invitrogen, USA). Quantitative PCR was performed using previously described methods^13^. Three replicates per sample were run in duplicate. The experimental cycle threshold (Ct) values were normalized to those obtained for GAPDH, and the relative gene expression level was determined using the 2_ddCT methods. Primer sequences were listed in the Supplementary Table S1.

### Generation of ARNT phosphorylation site mutant constructs

The phosphorylation site Ser77 on ARNT was changed to Aspartic acid by using QuikChange XL Site-Directed Mutagenesis Kit (Agilent, USA). The full-length mouse ARNT coding sequence (Origene, USA) was inserted into pGEM-T-Easy (Promega, USA) vector for a template DNA of site-directed mutagenesis. The following primers, which encompassed with mutated sites: Asp mARNT 5′-GAGCGGTTTGCCAGGGATGATGATGAG-3′ was used as a pair of complementary sense and antisense primer: mARNT 5′-TTTGTCATTACACATCTGGTCATC-3′ used for desired mutated construct. The PCR reaction was carried out according to the manufacturer’s protocol and thermal cycling condition was initiated with a denaturating step at 95 °C for 5 min followed by 35 cycles containing 95 °C for 50 s, 60 °C for 50 s, 68 °C for 6 min 30 s and a final extension at 68 °C for 7 min. The PCR product was then incubated with Dpn1 restriction enzyme for 2 h at 37 °C to remove the wildtype template from the reaction and subsequently transformed into XL10-Gold Ultracompetent Cells (Agilent Technologies, USA). The desired mutant coding sequences were then digested with AsisI/MluI restriction enzymes and subcloned into the modified PCMV6-EGFP (Origene, USA) and PCMV6-myc (Origene, USA) vector. The mutated constructs were carefully sequenced to confirm that mutagenesis was located in the proper position.

### Protein immunoprecipitation and MALDI-TOF mass spectrometry investigation of ARNT dimerization

To investigate whether the LB100 treatment favors ARNT dimer formation, we combined immunoprecipitation enrichment with MALDI-TOF mass spectrometry. For this purpose, endogenous ARNT was pulled down from MCT cell extracts using ARNT (5537, Cell Signaling Technology, USA) antibody. 8 × 10^6^ MCT cells have been used for each reaction. The binding proteins were eluted using 0.3% TFA and dried in speed vacuum centrifuge. For the mass spectrometric analysis, the samples were resuspended in 0.1% TFA and mixed with a saturated solution of the matrix sinapinic acid. After crystallization of the samples with the matrix, the measurement of the molecular weight of the immunoprecipitated proteins was carried out on a Rapiflex-MALDI-TOF-TOF mass spectrometer (Bruker, Germany) on linear positive mode.

### Phosphopeptides enrichment and mass spectrometric analysis

To target ARNT-phosphorylated peptide(s), an enrichment of the protein was carried out by immunoprecipitation using ARNT (5537, Cell Signaling Technology, USA) antibody and the Dynabeads Protein G (Thermo Fisher Scientific, Germany). After coupling the antibody to the beads, the samples were added, and the immunoprecipitation was carried out overnight at 4 °C under rotation. The washing step was performed using TBST and the binding ARNT was eluted according to the manufacturer recommendation. The samples were then dried in speed vacuum centrifuge. The eluted proteins were subjected to denaturation and NuPAGE SDS-PAGE (Novex Life technologies, USA) separation. The gels were stained with coomassie blue, and the visualized protein lanes were excised in the area of interest in 6 band sections each and subjected to in-gel-digestion with trypsin overnight at 37 °C. After extraction of the tryptic digests, the resulting peptides were subjected to purification step on a C18 Stage Tip. For phosphopeptide analysis, the purified peptide digest samples were subjected to TiO_2_ based phosphopeptide enrichment using the High-Select TiO_2_ kit (Thermo Fisher Scientific, Germany) following the manufacturer’s protocol. After enrichment, the resulting phosphopeptide fractions were reconstituted and 2 µl of the samples were injected for a single run on the column for LC–MS/MS analysis. The peptide sequence analysis was carried out on a hybrid quadruple-orbitrap mass spectrometer (Q Exactive, Thermo Fisher Scientific, Germany), and the tandem mass spectra were extracted. Phosphorylation of peptides identified as phosphopeptides via database searching was confirmed by manual inspection of the corresponding MS/MS spectra.

### Statistical analysis

ANOVA was used for multiple comparisons of groups to determine significance. Student's t-test analysis was used for the single-parameter comparisons. Statistical significance was defined as values of *p* < *0.05*, indicated as *p < 0.05, **p < 0.01, ***p < 0.001, ****p < 0.0001, NS *no significance*. GraphPad Prism 9.2.0 (283) software (GraphPad, La Jolla, USA) was used for statistical analysis.

### Schematic diagrams

The schematic of ARNT protein (Fig. 2b) was adapted and modified from literature and reanimated using Adobe Illustrator 25.4.1 (GunsmaaNyamsuren1)^22,23^.

## Supplementary Information

Supplementary Information.
