# Supplementary material for: PP2A phosphatase inhibition is anti-fibrotic through Ser77 phosphorylation-mediated ARNT/ARNT homodimer formation
Source: Sci Rep. 2021 Dec 15;11:24075. doi: 10.1038/s41598-021-03523-1 (PMC8674365; doi:10.1038/s41598-021-03523-1)
Supplement: Supplementary file 1 — Supplementary Information. [file 41598_2021_3523_MOESM1_ESM.pdf]

SUPPLEMENTARY INFORMATION

PP2A Phosphatase Inhibition is Anti-Fibrotic through Ser77 Phosphorylation-mediated ARNT/ARNT Homodimer Formation

Gunsmaa Nyamsuren[1,+], Gregor Rapp[1,+], Hassan Dihazi[1], Elisabeth M. Zeisberg[2,3], Desiree Tampe[1], Björn Tampe[1,+,*] Michael Zeisberg[1,3,+,*]

[1]*Department of Nephrology and Rheumatology, Göttingen University Medical Center, Georg August University, Göttingen, Germany*

[2]*Department of Cardiology and Pneumology, Göttingen University Medical Center, Georg August University, Göttingen, Germany*

[3]*German Center for Cardiovascular Research (DZHK), Robert Koch Street 40, Göttingen, Germany*

[+]These authors contributed equally to this work

*Corresponding authors

Correspondence:

Michael Zeisberg, MD
Department of Nephrology and Rheumatology
Göttingen University Medical Center
Georg August University
Göttingen, Germany
Email: michael.zeisberg@med.uni-goettingen.de

or

Björn Tampe, MD
Department of Nephrology and Rheumatology
Göttingen University Medical Center
Georg August University
Göttingen, Germany
Email: bjoern.tampe@med.uni-goettingen.de

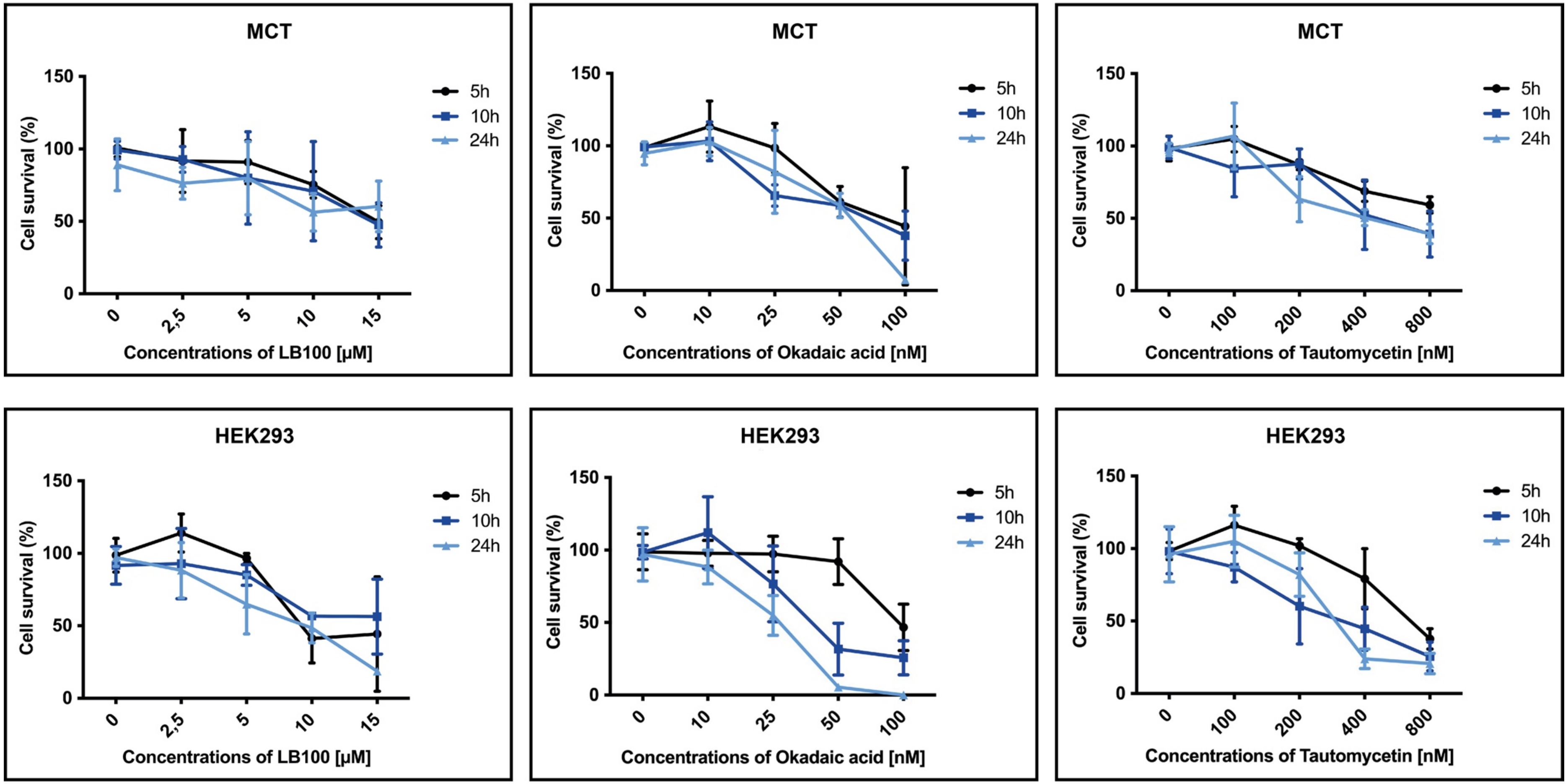

Fig. S1. Cytotoxicity of different drugs. Cell survival was examined by the MTT assay for establishing the drug concentration or applying time point in MCT and HEK293 cells. The average mean of the results was obtained from four independent experimental replicates.

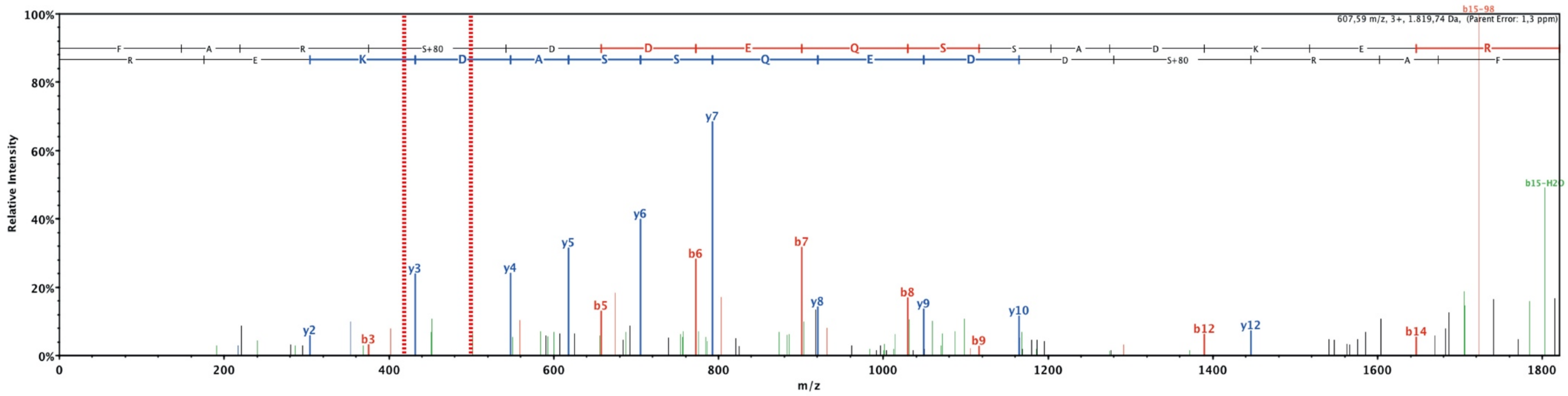

Fig. S2. Identification of ARNT phosphorylation site. MS/MS spectrum was generated using Scaffold (v4.10.0) (http://www.proteomesoftware.com/products/scaffold). MS/MS spectrum of the three times charged precursor ion at m/z 1819.74 observed in the phosphopeptide-enriched fraction from ARNT immunoprecipitation digests. The peptide is identified as the phosphopeptide FAR**pS**DDEQSSADKER. The phosphorylation site is unambiguously identified at the serine on position 4 of the peptide (Ser77 in ARNT).

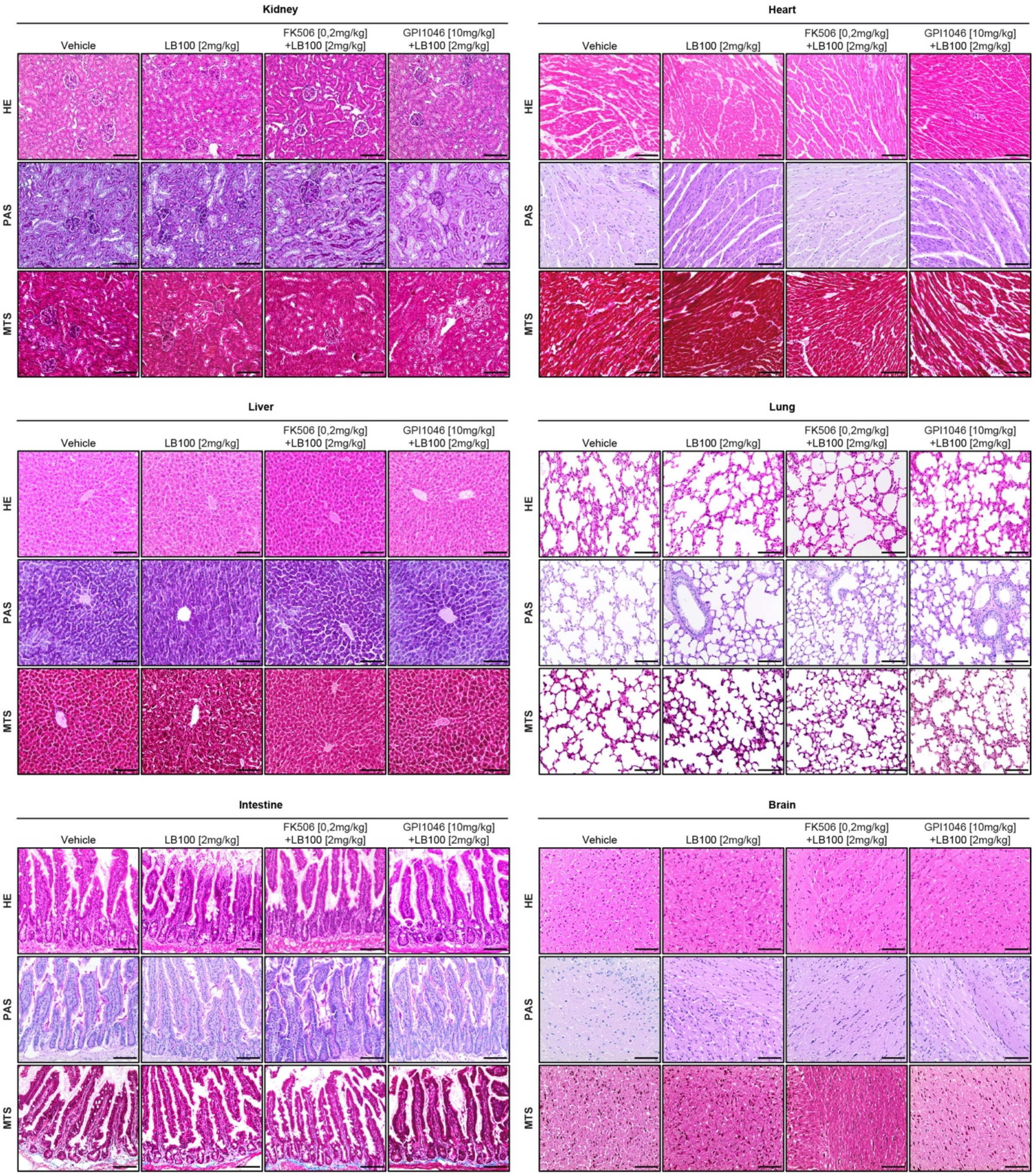

Fig. S3. No severe inflammations or obvious tissue damages were observed in mice exposed to LB100 treatment. Representative light microscopy images of HE stained main organs (kidney, heart, liver, lung, intestine and brain) of UUO challenged mice that treated with indicating treatment. n=6/group. Scales bars: 25 μm.

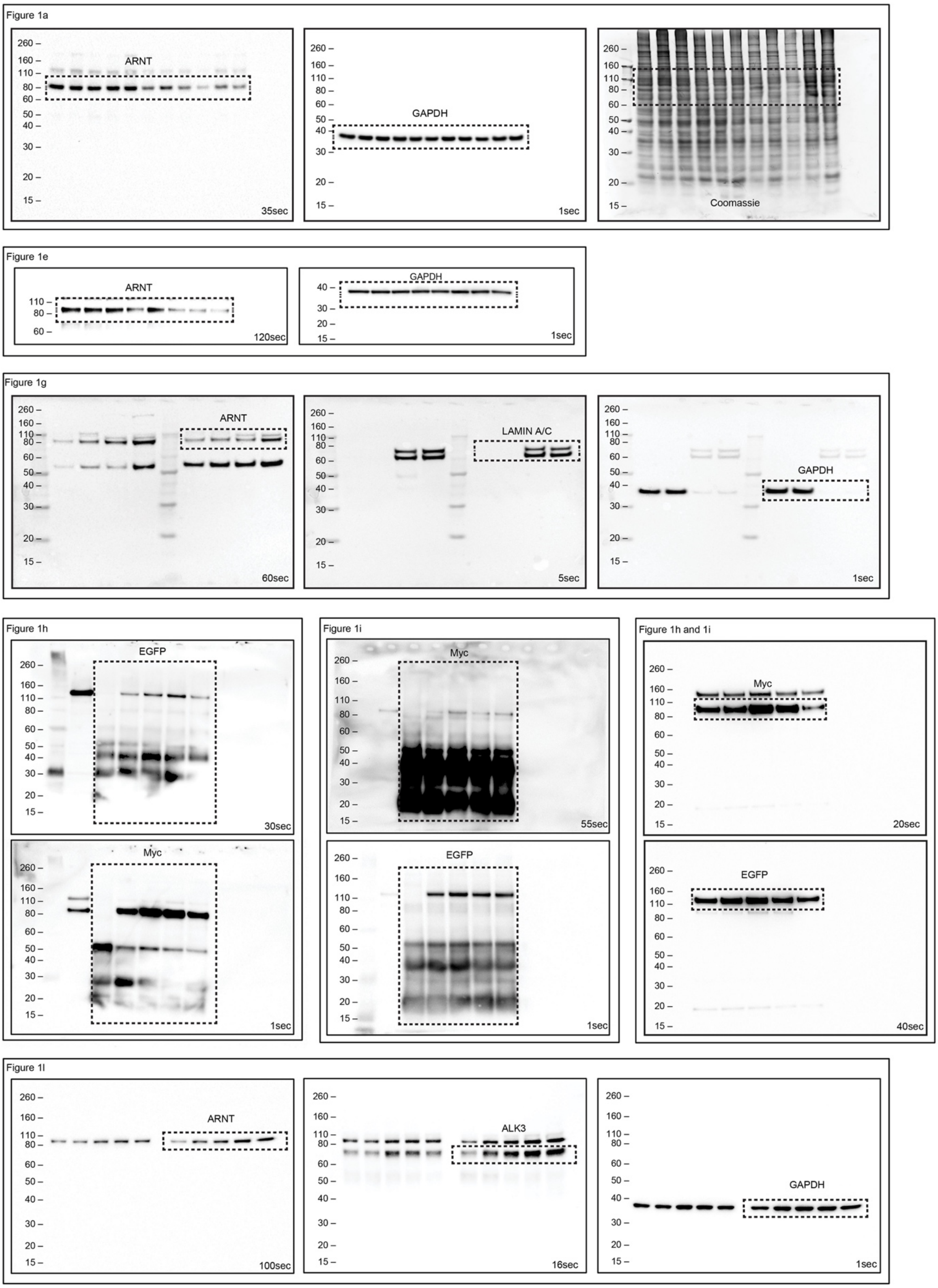

Fig. S4. Uncropped full-length images of western blotting membranes. Boxes indicate selected areas in the main Fig. 1a, e, g, h, i and l. Membranes were often stripped and reprobed for multiple antibodies.

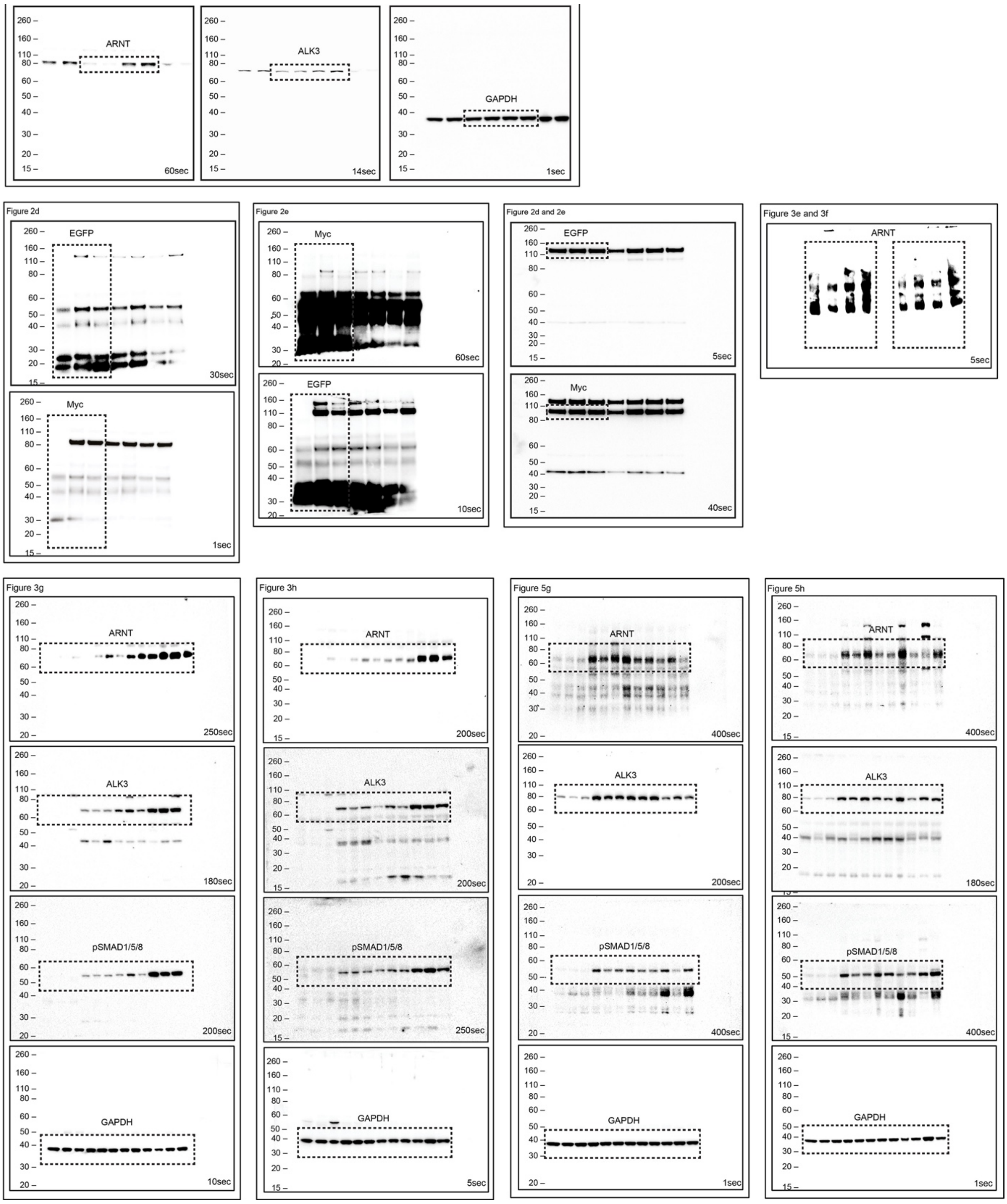

Fig. S5. Uncropped full-length images of western blotting membranes. Boxes indicate selected areas in the main Fig. 1n, 2d, 2e, 3e, 3f, 3g, 3h, 5g and 5h. Membranes were often stripped and reprobed for multiple antibodies.

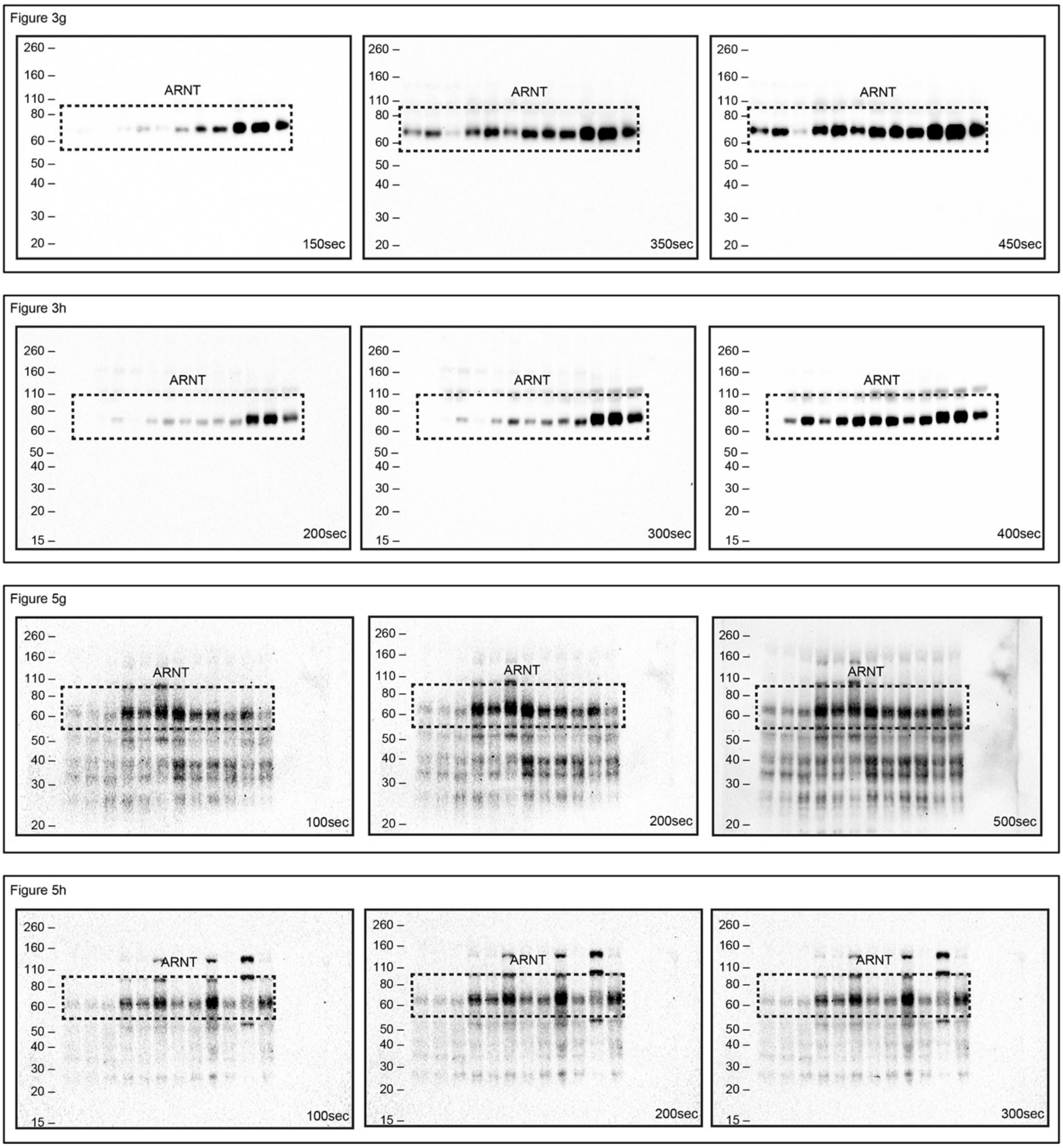

Fig. S6. Different exposure images of western blotting membranes. Boxes indicate selected areas in the main Fig. 3g, 3h, 5g and 5h. Three different exposures are shown.

Primer	Sequence
mouse ALK3-F	5’-TGGCACTGGTATGAAATCAGAC-3’
mouse ALK3-R	5’-CAAGGTATCCTCTGGTGCTAAAG-3’
mouse ARNT-F	5’-TCTCCCTCCCAGATGATGAC-3’
mouse ARNT-R	5’-CAATGTTGTGTCGGGAGATG-3’
mouse GAPDH-F	5’-TGACCTCAACTACATGGTCTACA-3’
mouse GAPDH-R	5’-CTTCCCATTCTCGGCCTTG-3’

Table. S1. List of the primer sequences used for qRT-PCR.

Antibody	Species	Dilution for WB	Dilution for IF	Catalog Number	Source
ALK3	rabbit, polyclonal	1:1000		38-6000	Invitrogen
αSMA (1A4)	mouse, monoclonal		1:500	A5228	Sigma
ARNT (D28F3)	rabbit, monoclonal	1:1000	1:100	5537	Cell Signaling Technology
Collagen-1	goat		1:10	1310-30	Southern Biotech
EGFP (GF28R)	mouse, monoclonal	1:1000		MA5-15256	Invitrogen
GAPDH (6C5)	mouse, monoclonal	1:8000		sc-32233	Santa Cruz
Lamin A/C (4C11)	mouse, monoclonal	1:4000		4777	Cell Signaling Technology
myc-tag (9B11)	mouse, monoclonal	1:1000		2276	Cell Signaling Technology
pSmad158 (D5B10)	rabbit, monoclonal	1:1000		13820	Cell Signaling Technology

Table. S2. List of the antibodies.
